# Systemic microvascular rarefaction is correlated with dysfunction of late endothelial progenitor cells in mild hypertension: a substudy of EXCAVATION-CHN1

**DOI:** 10.1186/s12967-019-2108-8

**Published:** 2019-11-12

**Authors:** Jianwen Liang, Yan Li, Long Chen, Wenhao Xia, Guifu Wu, Xinzhu Tong, Chen Su, Jiang He, Xiufang Lin, Jun Tao

**Affiliations:** 1 Department of Hypertension and Vascular Disease, The First Affiliated Hospital, Sun Yat-Sen University, Guangzhou, 510080 China; 2 Department of Cardiology, The Eighth Affiliated Hospital, Sun Yat-Sen University, Shenzhen, China; 3 Department of Cardiology, The Fifth Affiliated Hospital, Sun Yat-Sen University, Zhuhai, 519000 China

**Keywords:** Hypertension, Microcirculation, Microvascular rarefaction, Optical coherence tomography angiography, Late endothelial progenitor cells

## Abstract

**Background:**

Hypertension often presents with microvascular rarefaction (MVR), which is closely associated with impaired angiogenesis. Early detection of MVR is essential for systemic assessment in patient with hypertension. We aimed to determine the systemic MVR through both optical coherence tomography angiography (OCTA) and intravital capillaroscopy, and to investigate their respective efficacies and related mechanisms associated with late endothelial progenitor cells (LEPCs) dysfunction.

**Methods:**

Seventy-one hypertensive and sixty-nine age-match normotensive subjects were included in this study. All subjects received intravital capillaroscopy for skin capillary density (SCD) and OCTA for retinal capillary density (RCD) and non-perfused areas (R-NPA). Subsequently, correlation of LEPCs activities and microvascular rarefaction were examined.

**Results:**

Compared with normotensive subjects, hypertensive patients had significantly lower RCD [(52.9 ± 2.9)% vs. (57.8 ± 1.6)%, *P *< 0.01] and higher R-NPA [(0.12 ± 0.07) mm^2^ vs. (0.053 ± 0.020) mm^2^, *P *< 0.01]. SCD correlated positively with RCD but negatively with R-NPA [(RCD: OR = 0.40, 95% CI 0.25–0.67, *P *< 0.01); (R-NPA: OR = 0.39, 95% CI − 0.0029 to 0.0011, *P *< 0.01)]. The discriminative powers of RCD performed best (AUC 0.79 versus SCD AUC 0.59, P < 0.001) followed by R-NPA (AUC 0.73 versus SCD AUC 0.59, P < 0.001) for systolic blood pressure. Similar pattern is also found for diastolic blood pressure (RCD AUC 0.80 versus SCD AUC 0.54, P < 0.001; R-NPA AUC 0.77 versus SCD AUC 0.54, P < 0.001). Furthermore, LEPCs tube formation was impaired in hypertensive patients (36.8 ± 2.3 vs. 28 ± 3.7, *P *< 0.01). After multivariate adjustments, positive correlation existed between RCD or R-NPA with LEPCs tube formation (RCD: β = 0.64, 95% CI 0.34–0.91, *P *< 0.01; R-NPA: β = − 24.67, 95% CI − 43.14 to − 4.63, *P *< 0.05) but not with SCD (β = 0.082, 95% CI 0.01–0.18, *P *= 0.085).

**Conclusion:**

Compared to intravital capillaroscopy, OCTA is a more precise technique for early detection of hypertensive microvascular rarefaction, which is associated with the fall in LEPC-mediated angiogenesis. Both of OCTA and LEPCs function can help identify hypertension-related capillary abnormality.

*Trail Registration* The trial is a substudy of EXCAVATION-CHN1, registered at clinicaltrials.gov as NCT02817204 (June 26, 2016).

## Background

Hypertension is one of major contributing factors to the development of atherosclerotic cardiovascular diseases (ASCVD) [1]. Recent advances have shed light on the hallmark of hypertension-related microvascular deficiency [2, 3]. Microvascular rarefaction (MVR), a loss of terminal arterioles and capillaries, is generally regarded as a pivotal pathophysiological process of hypertension in its early stage [4]. Early and effective detection of MVR is crucial for hypertensive management. However, the commonly used technique, namely, intravital capillaroscopy, is largely limited by its low accuracy and reproducibility [5], and is thus unsatisfactory for describing systemic microvascular circulation. Besides, it has been reported that MVR is closely associated with impaired angiogenesis [6–8]. Since endothelial progenitor cells (EPCs) is essential for angiogenesis in hypertension [9], and impaired function of EPCs was observed in patients with hypertension [10, 11], it is plausible to postulate a mechanistic link between microcirculation and angiogenesis status in hypertension that warrants further investigations.

Recently, optical coherence tomography angiography (OCTA) gradually emerges as a new technique for the evaluation of retinal microcirculation in clinical practice [12, 13]. With the advantages of relatively fast working speed, operating security and non-invasion, OCTA is able to provide more quantitative measurements of retinal non-perfusion area (R-NPA), and offer better visualization of retinal capillary density (RCD) in capillary layers [14]. Current researches showed OCTA provides more specific and sensitive assessment for diabetic retinopathy [5]. It has been long recognized that capillary-related ocular vasculature deficiency is an early marker of hypertensive pathophysiology. Therefore, accurate evaluation of retinal microcirculation has always been an attractive option for the early and effective identification of hypertension-related microvascular rarefaction. To date, few data have been reported about retinal MVR detection with OCTA in patients with hypertension.

EPCs is a specialized subset of hematopoietic cells found in the adult bone marrow and peripheral circulation arising from hemangioblasts prenatally. EPCs are substantially crucial for the preservation of a structurally and functionally intact endothelium and the maintenance of the normal endothelial integrity [15, 16]. EPCs are divided into early (EEPCs) and late outgrowth EPCs (LEPCs) [17]. EPCs play a key role in the homeostasis between MVR and angiogenesis in hypertension and it has been shown that LEPCs are superior to EEPCs with regard to angiogenesis in hypertension [9]. Thus, based on our previous finding in connection between EEPCs and vascular repairmen in hypertensive patients, we proposed that diminished LEPCs may in some way affect angiogenesis in hypertensive MVR and that its dysfunction is associated with the presence of MVR in hypertension. However, the relationship between MVR and LEPC dysfunction in hypertension remains unknown.

In light of these, we hypothesized that OCTA can identify hypertensive MVR in an early phase, as observed by conventional capillaroscopy, and that microvascular abnormality may be correlated with LEPCs dysfunction, as suggested by previous observations. Also, we would like to find out which method of detection is superior for early detection of hypertensive microvascular rarefaction. Therefore, we enrolled hypertensive patients and age-matched normotensive volunteers. First, we investigated whether OCTA can effectively detect MVR in mild hypertension; second, the correlation between OCTA and traditional intravital capillaroscopy, and the association of retinal microvascular perfusion and blood pressure were compared; last, LEPCs function were tested and the association between LEPC-mediated angiogenesis and MVR was examined.

## Methods

### Study design

Microvascular rarefaction plays a key role in hypertension-induced microvascular organ damage which is associated with high morbidity and mortality. Dysfunction of endothelial progenitor cells (EPCs) leads to impaired angiogenic capacity in hypertension contributing to capillary rarefaction. In order to identify whether aerobic exercise can improve angiogenesis capacity of EPCs which contributes to ameliorate microvascular rarefaction in hypertension, EXCAVATION-CHN1 was designed and registered as NCT02817204, which was a single-center, open-label, controlled, parallel group study conducted in Guangzhou, Guangdong, China. Previous to perform exercise intervention, it is necessary to identify the status of the microvasculature and EPCs functions between normotension and hypertension.

This study is the sub-study of RCT EXCAVATION-CHN1. Seventy-one essential hypertensive patients without any treatment for high blood pressure and sixty-nine normotensive subjects matched for age were enrolled, based on the inclusion criteria of EXCAVATION-CHN1: aged 25 to 40 years; blood pressure raised with home blood pressure (HBP) 140 mmHg to 159 mmHg and/or diastolic blood pressure 90 mmHg to 100 mmHg; non-smoker; no previous exposure to anti-hypertensive medication, herbal supplements, anti-depressants or other traditional Chinese medication. In addition, the rationale for exclusive male enrollment was based on the consideration that menstruation would affect the completion of the continuous exercise training protocol (5 times a week and lasts for 12 weeks) in female subjects. Other key exclusion criteria included secondary hypertension; history or presence of cardiovascular disease; diabetes or pre-diabetes mellitus or systemic immune diseases.

The study consisted of two phases: first, a 2-week run-in period through HBP monitoring and baseline blood biochemical examination; second, 1-week intravital capillaroscopy and OCTA examination.

### Blood pressure

BP measurement was performed in accordance with the method recommended by clinical guidelines [18, 19]. HBP consisted of BP measurements twice a day, one between 6:00 a.m. and 9:00 a.m. and the other between 6:00 p.m. and 9:00 p.m. Before the measurements, subjects were required to refrain from caffeine and alcohol for at least 2 h. Having rested for at least 5 min after their arrival at the test site, subjects were examined while seated in a quiet and temperature-controlled room, with the feet on the floor and arm supported at heart level. BP was measured by an automated device (OMRON, HEM-7133, Japan) and by at least two measurements spaced 2 min apart. Additional measurements were to be taken if the first two measurements were significantly different.

### Intravital capillaroscopy

The intravital capillaroscopy was performed with final 136× magnification on 19-inch display (Videocapillaroscopy: XS3000, JIANNENG Optical Instrument Lmt.com, An Hui, China; Display: Phillips 19S4QAB, Suzhou, China). The skin of the dorsum of the middle phalanx of the left hand was examined. Four microscopic fields (0.66 mm^2^ per field) centered on an ink spot at each site were recorded continuously for 5 min to detect intermittently perfused capillaries, images and videos were coded and stored with Videocap 8.14 software (DS-Medical, Milan, Italy). Capillary density in the resting state was counted during a 15-second period, during which visible perfused capillaries (continuously and intermittently) were counted. We applied venous congestion (VC), with the digital cuff (OMRON, HEM-7131, Japan) inflated to 60 mmHg for 2 min, to expose a maximal number of non-perfused capillaries. Using the same visual fields that were used during resting state, the capillaries were counted in the 2-min recordings.

Skin capillary density (SCD) was counted offline to determine whether a capillary was present or not by a single-experienced investigator who was blinded to the group assignments from a freeze-framed reproduction of the videotape and from the running videotape, as well as the hypertensive status and blood pressure data of the study subjects.

### Optical coherence tomography angiography (OCTA)

OCTA was performed using OptovueAngioVue system (RTVue XR Avanti, Optovue, Inc., Fremont, CA, U.S). After the subject was seated, the operator used joystick to move optic head with the chinrest up and down. Adjusted the mark in the IR live video on the monitor to center subject’s pupil of oculus dexter (OD) or oculus sinister (OS), moved forward slowly and asked subject to focus on the blue fixation target inside the machine, then stopped the machine until the retinal IR image reached sharpness, and executed the auto adjustment. When the adjustment finished, the operator started scanning by using a light source centered on 840 nm and bandwidth of 50 nm in order to a high-quality OCT images on the monitor. With split-spectrum amplitude-decorrelation angiography (SSADA) technique [13], each OCTA volume contained 304 × 304 A-scans with two consecutive B-scans captured at each fixed position before proceeding to the next sampling location.

RCD and R-NPA measurements were performed on 3 mm × 3 mm retinal parafovea scan field size and were used to assess ocular perfusion impairment in vascular conditions. RCD was a measurement of the proportion of pixels occupied by flowing vessels out of the total pixels within the area of analysis (Software Version 2015.1.0). The outputs of RCD analysis included color-coded vessel map and regional flow density map. R-NPA was obtained by the software, while the outputs were the non-perfused areas by mouse click selection. Ischemic areas would be shown in yellow and be saved and matched with others in the study [14]. OD and OS mean values of RCD and R-NPA were calculated for further analysis.

### LEPCs culture and characterization

Late EPCs were cultured and characterized as in our previous study [20]. Briefly, PBMCs from healthy subjects were cultured on fibronectin-coated 6-well plates in EBM-2 supplemented with endothelial growth medium-SingleQuots (Clonetics, San Diego, CA, USA). After a 4-day culture, non-adherent cells were removed by thoroughly washing with culture medium. Medium was changed daily for 7 days, and then every other day until the first passage. For all assays, LEPCs were used at passages 3 (about 28 days). After 28 days of culture, marker proteins of cultured EPCs were examined by flow cytometry analysis using phycoerythrin (PE)-labeled monoclonal mouse anti-human antibodies recognizing CD31 (BD Pharmingen), Tie-2 receptor (BD Pharmingen) and kinase-insert domain receptor (KDR) (R&D system). Overall, (92.25 ± 4.8)% of the cells were positive for CD31, (80.16 ± 6.5)% for KDR, and (88.36 ± 5.6)% for Tie-2 receptor. Based on the isolation and cultivation protocol, the adherent mononuclear cells were identified as late EPCs.

### LEPCs migration in vitro

LEPCs migration was determined using a modified Boyden chamber. Briefly, 2 × 10^4^ EPCs, resuspended in 250 µl EBM-2, were pipetted in the upper chamber of a modified Boyden chamber (Costar Transwell^®^ assay, 8 µm pore size, Corning, NY). The chamber was placed in a 24-well culture dish containing 500 µl EBM-2 supplemented with either PBS, or 100 ng/ml SDF-1 (Peprotech, Rocky Hill, NJ, USA). After 24-h incubation at 37 °C, transmigrated cells were counted by independent investigators blinded to treatment groups.

### LEPCs adhesion in vitro

A monolayer of human umbilical vein endothelial Cells (HUVECs) was prepared 48 h before the assay by plating 2 × 10^5^ cells in each well of a 4-well plate. HUVECs were pretreated with or without 1 ng/ml tumor necrosis factor-α (TNF-α, Peprotech) for 12 h. Then 1 × 10^5^ CM-DiI (CellTracker™ CM-DiI, Invitrogen)-labeled EPCs were added to each well and incubated for 3 h at 37 °C. Nonattached cells were gently removed with PBS, and adherent EPCs were fixed with 4% paraformaldehyde and counted by independent investigators blinded to treatment groups.

### LEPCs tube formation in vitro

LEPCs tube formation experiment was conducted as follows: a growth factor-reduced Matrigel (Corning) was warmed up at 4 °C overnight. After completely thawed, 60 μl of Matrigel was plated to 96-well plates at the same level to distribute evenly, and incubated for 1 h at 37 °C. LEPCs (2 × 10^4^) were resuspended with EBM-2, and loaded on the top of the Matrigel. Each conditional group contained 3 wells. Following incubation at 37 °C for 2 h, each well was imaged directly under a microscope, and an average of tubules was counted from 3 to 5 random fields.

### Statistical analysis

A formal sample size calculation was not possible, because of the lack of pre-existing data. A sample size of 10 healthy volunteers was chosen for the normotensive cohort to allow detection of a single SD [21] change in levels of RCD. Thus, we had a power of 80% to detect a difference of 30–40% in the primary parameter between groups.

The statistical analysis was performed by an independent medical statistician. Continuous variables were described as the mean ± SD for normally distributed data (baseline clinical characteristics, SCD, RCD, R-NPA and LEPCs tube formation). Comparisons between groups were performed with Student *t* test (normally distributed data). Correlations between variables (SCD and RCD or R-NPA) were analyzed by linear regression analysis. With adjustment for confounding factors [age, body mass index (BMI), dietary salt intake, mean systolic blood pressure (MSBP), mean diastolic blood pressure (MDBP), triglycerides (TG), total cholesterol (TC), low-density lipoprotein (LDL), fasting blood glucose (FBG)], since the dependent variables are continuous variables, and the independent variables basically conform to the LINE criteria (linear, independent, normal distribution, equal variance), we adopted the multivariant linear regression model with beta value and 95% confidence interval (CI) to determine which factors were the independent predictors of LEPCs tube formation ability.

In our study, SBP and DBP data is normally distributed. The SBP (132 ± 6.2) mmHg and DBP (80 ± 5.6) mmHg were divided into binary variable, and the classification of SBP and DBP by SCD, RCD and R-NPA was evaluated by receiver operating characteristic curve (ROC) method, using the area under the curve (AUC) as a criterion for classification. All tests were 2-tailed, and a statistical significance was established at *P *< 0.05. Analysis was performed using SPSS software (version 22.0; SPSS, Inc., Chicago, IL) and figures were plotted using Prism software (version 6.0).

## Results

### Baseline characteristics

A total of 160 individuals were screened. 140 individuals successfully completed the run-in period and were assigned to either hypertensive or normotensive group. The primary reason for discontinuation before assignment was failure to meet all inclusion/exclusion criteria. As summarized in Table 1, among all the subjects, mean age was 36.37 years, mean BMI was 24.6 kg/m^2^ and the proportion of obese individuals (BMI 30 kg/m^2^). No statistically significant differences between groups were found in the main demographic characteristics including serum glucose level, dietary salt intake (obtained by self-monitoring continuously for 5 days), serum lipid level and renal function. However, BP level between normotensive and hypertensive group was significant differences [(SBP: 125.1 ± 7.7 mmHg vs. 143.6 ± 7.4 mmHg, *P *< 0.001); (DBP: 73.2 ± 5.56 mmHg vs. 89.0 ± 5.8 mmHg, *P *< 0.001)] (Table 1).

**Table 1:** Baseline characteristics of the all population

	Normotension	Hypertension	*t*	*P*
Total, n	69	71		
Age, year	36.17 ± 4.10	36.6 ± 5.60	0.52	0.61
Gender, male%	100	100		
Weight, kg	73.20 ± 5.50	73.0 ± 11.30	0.13	0.89
BMI, kg/m^2^	24.40 ± 1.40	24.8 ± 3.00	1.00	0.32
Daily salt intake, g/day	5.31 ± 1.22	5.26 ± 0.95	0.27	0.79
FBG, mmol/L	4.76 ± 0.90	4.7 ± 0.40	0.51	0.61
Fasting TC, mmol/L	3.90 ± 0.40	3.8 ± 0.70	1.03	0.30
Fasting HDL, mmol/L	1.10 ± 0.10	1.2 ± 0.20	1.31	0.19
Fasting LDL, mmol/L	2.90 ± 0.30	3.0 ± 0.70	1.09	0.28
Fasting TG, mmol/L	1.50 ± 0.50	1.4 ± 0.60	1.07	0.29
Creatinine, mcmol/L	79.10 ± 11.60	78.2 ± 11.20	0.47	0.62
CRP, mmol/L	1.00 ± 0.50	1.5 ± 1.70	2.33	0.02
Home blood pressure				
SBP, mmHg	125.1 ± 7.7	143.6 ± 7.4	− 14.53	0.0001
DBP, mmHg	73.2 ± 5.56	89.0 ± 5.8	− 16.50	0.0001
Heart rate, bpm	73.2 ± 5.0	74.9 ± 8.1	− 1.48	0.14

All values are expressed as mean ± SD

*BMI* body mass index, *FBG* fasting blood glucose, *TC* total cholesterol, *HDL* high density lipoprotein, *LDL* low density lipoprotein, *TG* triglyceride, *CRP* C-reactive protein, *SBP* systolic blood pressure, *DBP* diastolic blood pressure

### Capillary density and microvascular perfusion of hypertension were impaired

**Fig. 1:**
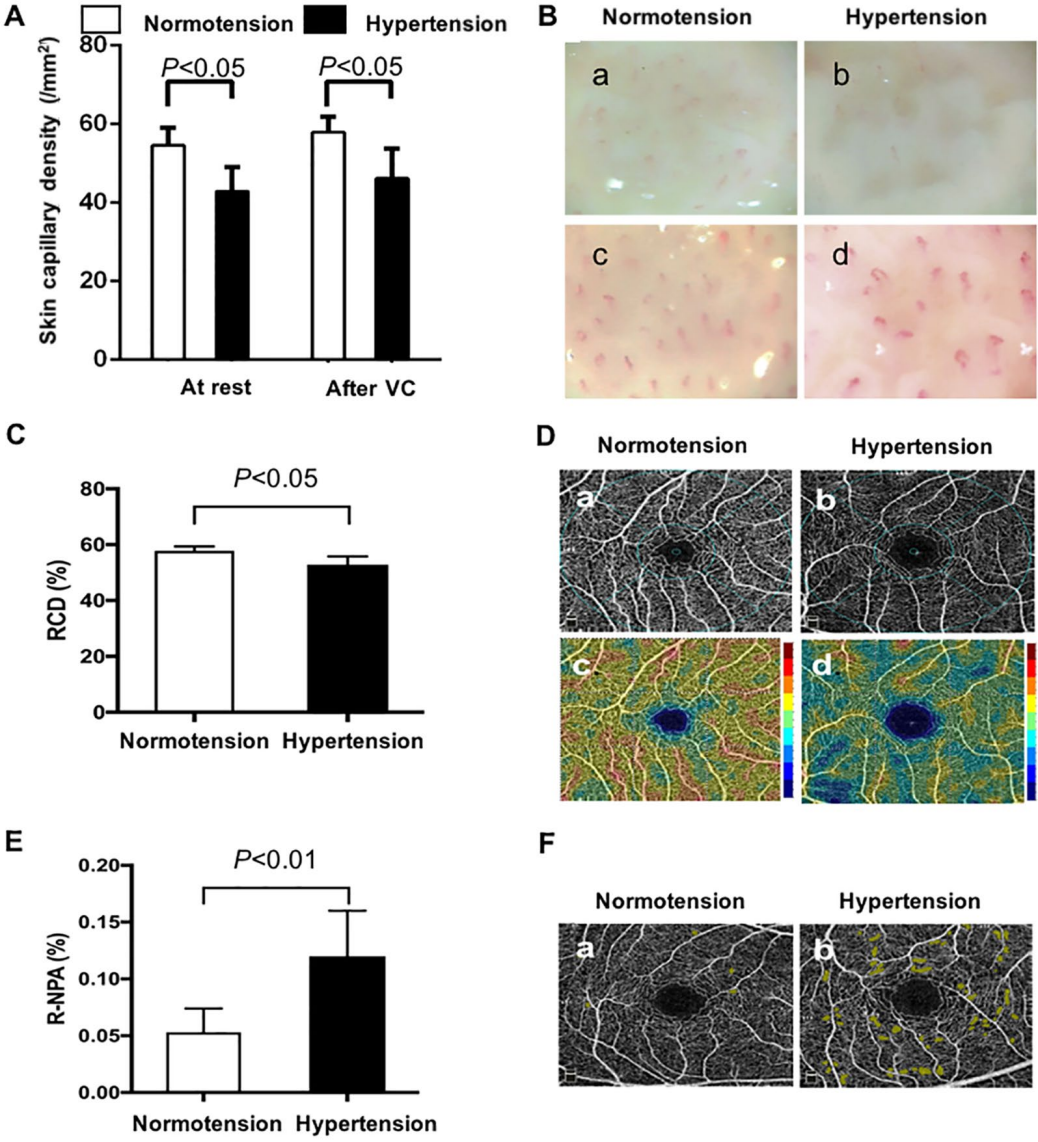
Quantification and representative photographs of SCD, RCD, R-NPA in normotension and hypertension. **A** Quantification of SCD at rest and after 2-min VC in both groups; **B** representative photographs of SCD at rest and after VC in both groups. Hypertensive SCD (b, d) was reduced than the normotensive (a, c) in the rest state and after 2-min VC; **C** quantification of RCD in both groups; **D** representative photographs of retinal vessel map and flow density map. Both hypertensive retinal vessel map (b) and flow density map (d) decreased than that in normotension (a, c); **E** quantification of R-NPA in both groups; **F** Representative photographs of R-NPA. Yellow zone represented non-perfused areas. Representative photographs of retinal NPA in hypertension (b) increased than that in normotension (a). (**B** 0.66 mm^2^ per field, magnification 136, scale bar 100 μm; **D** and **F** optical axial resolution, 5 microns; optical transverse resolution, 15 microns). *VC* venous congestion, *RCD* retina capillary density, *R-NPA* retinal non-perfused areas

SCD of hypertensive patients was lower than that of normotensive subjects in the resting state and after 2-min VC (both *P *< 0.05) (Fig. 1A, B); additionally, the increased density after VC showed no significant difference between groups (normotension (3.2 ± 7.9)/mm^2^ vs. hypertension (3.0 ± 5.7)/mm^2^, *P *> 0.05). RCD was decreased [(52.0 ± 6.5)% vs. (57.6 ± 1.6)%, *P *< 0.01] and R-NPA was increased in hypertensive patients [(0.12 ± 0.07) mm^2^ vs. (0.058 ± 0.026) mm^2^, *P *< 0.01] (Table 2; Fig. 1C–F).

**Table 2:** Microcirculation comparison of normotension and hypertension

	Normotension	Hypertension	*t*	*P*
Skin capillary density, /mm^2^				
In the rest state	54.9 ± 4.2	43.5 ± 5.8	13.25	0.0001
After 2-min VC	57.7 ± 3.9	46.3 ± 7.7	10.95	0.0001
Absolute increase NCD, %	3.2 ± 7.9	3.0 ± 5.7	0.17	0.86
RCD, %				
OD	57.9 ± 1.5	52.7 ± 2.5	14.82	0.0001
OS	57.7 ± 1.7	53 ± 3.3	10.5	0.0001
Mean	57.8 ± 1.6	52.9 ± 2.9	12.28	0.0001
R-NPA, mm^2^				
OD	0.053 ± 0.022	0.13 ± 0.079	− 7.76	0.0001
OS	0.051 ± 0.019	0.11 ± 0.062	− 7.52	0.0001
Mean	0.053 ± 0.026	0.12 ± 0.07	− 7.54	0.0001

*VC* venous congestion, *RCD* retina capillary density, *R-NPA* retinal non-perfused areas, *OD* oculus dexter, *OS* oculus sinister, *SD* standard deviation

### RCD and R-NPA showed a consistent tendency with classical SCD

**Fig. 2:**
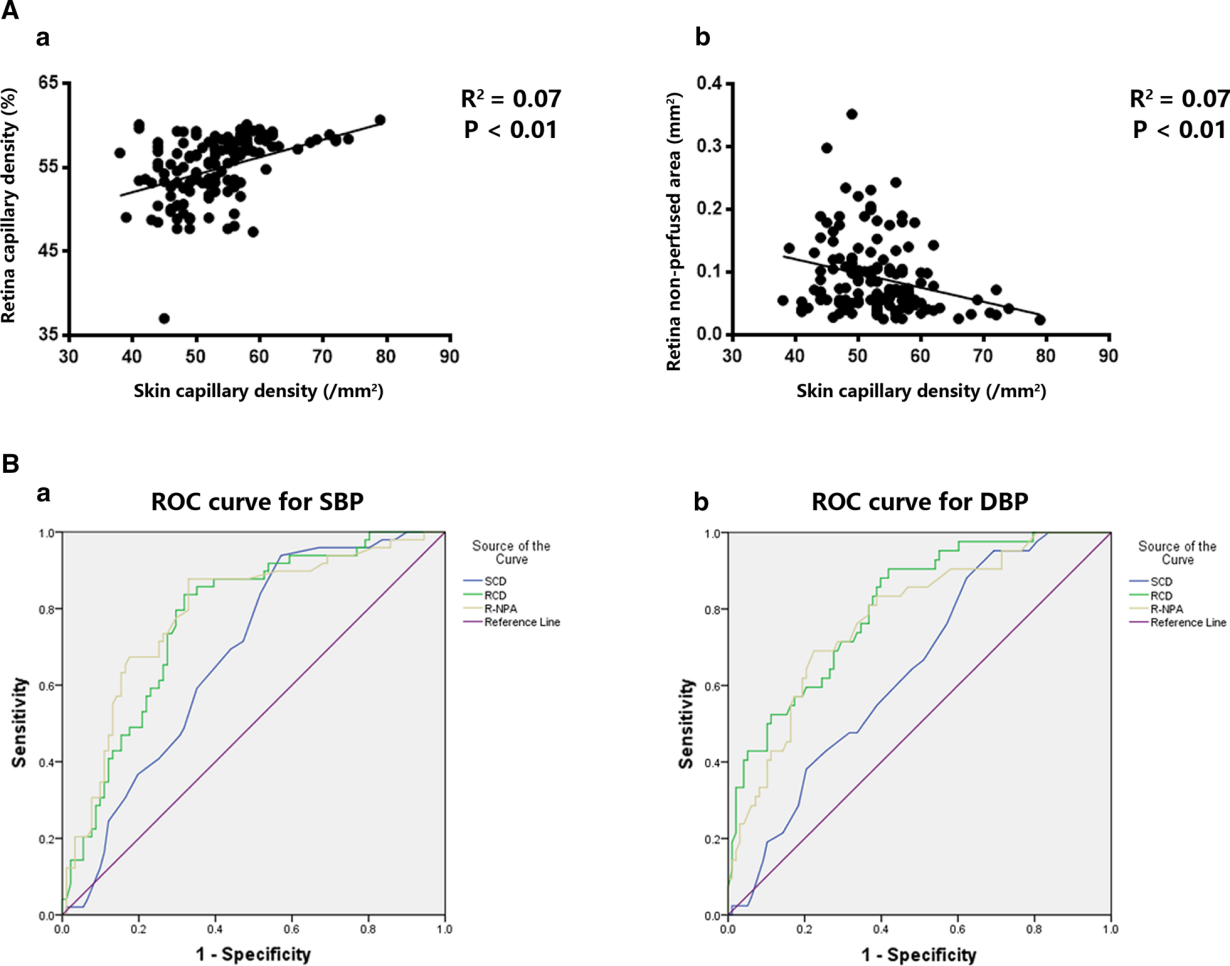
Correlation and receiver operating characteristic (ROC) analysis about results of intravital capillaroscopy and OCTA. **A** Scatter plots of correlation between RCD and SCD (a) and scatter plots of correlation between R-NPA and SCD (b). **B** ROC curve for SCD and RCD, R-NPA. ROC was performed for blood pressure (definition of high blood pressure), n = 140. a with SBP analysis, AUC for SCD is 0.68, 0.72 for RCD and 0.79 for R-NPA; b with DBP analysis, AUC for SCD is 0.64, 0.81 for RCD and 0.78 for R-NPA. *OCTA* optical coherence tomography angiography, *SCD* skin capillary density, *RCD* retina capillary density, *R-NPA* retina non-perfused areas, *AUC* area under the curve

To understand the extent of retinal capillary impairment of both normotensive and hypertensive patients, we investigated the relationship between SCD and RCD (mean of OD and OS), as well as between SCD and R-NPA (mean of OD and OS) because SCD by intravital capillaroscopy was the most classic and recognized approach. These data showed that SCD had moderate-intensity positive correlation with RCD and negative correlation with R-NPA [(RCD: R = 0.269, 95% CI 0.1074–0.4159, *P *< 0.01); (R-NPA: R = − 0.273, 95% CI − 0.4202 to − 0.1125, *P *< 0.01)] (Fig. 2A).

### RCD and R-NPA correlated better with blood pressure than SCD

Patients with hypertension presented with lower RCD and higher R-NPA; however, it remains unknown whether or not the changes of retinal OCTA were correlated with blood pressure level compared with the traditional SCD. In this study, the discriminative powers of SCD, RCD and R-NPA of SBP and DBP were compared by their respective ROC curves. As is shown in Fig. 2Ba, RCD perform best (RCD AUC 0.72 vs. SCD AUC 0.68, *P* < 0.001) followed by R-NPA (R-NPA AUC 0.79 vs. SCD AUC 0.68, *P* < 0.001) in SBP analysis. Similar pattern is also found in DBP analysis (RCD AUC 0.81 versus SCD AUC 0.64, *P *< 0.001; R-NPA AUC 0.78 versus SCD AUC 0.64, *P *< 0.001) (Fig. 2Bb).

### LEPC-mediated angiogenesis was declined in hypertension

**Fig. 3:**
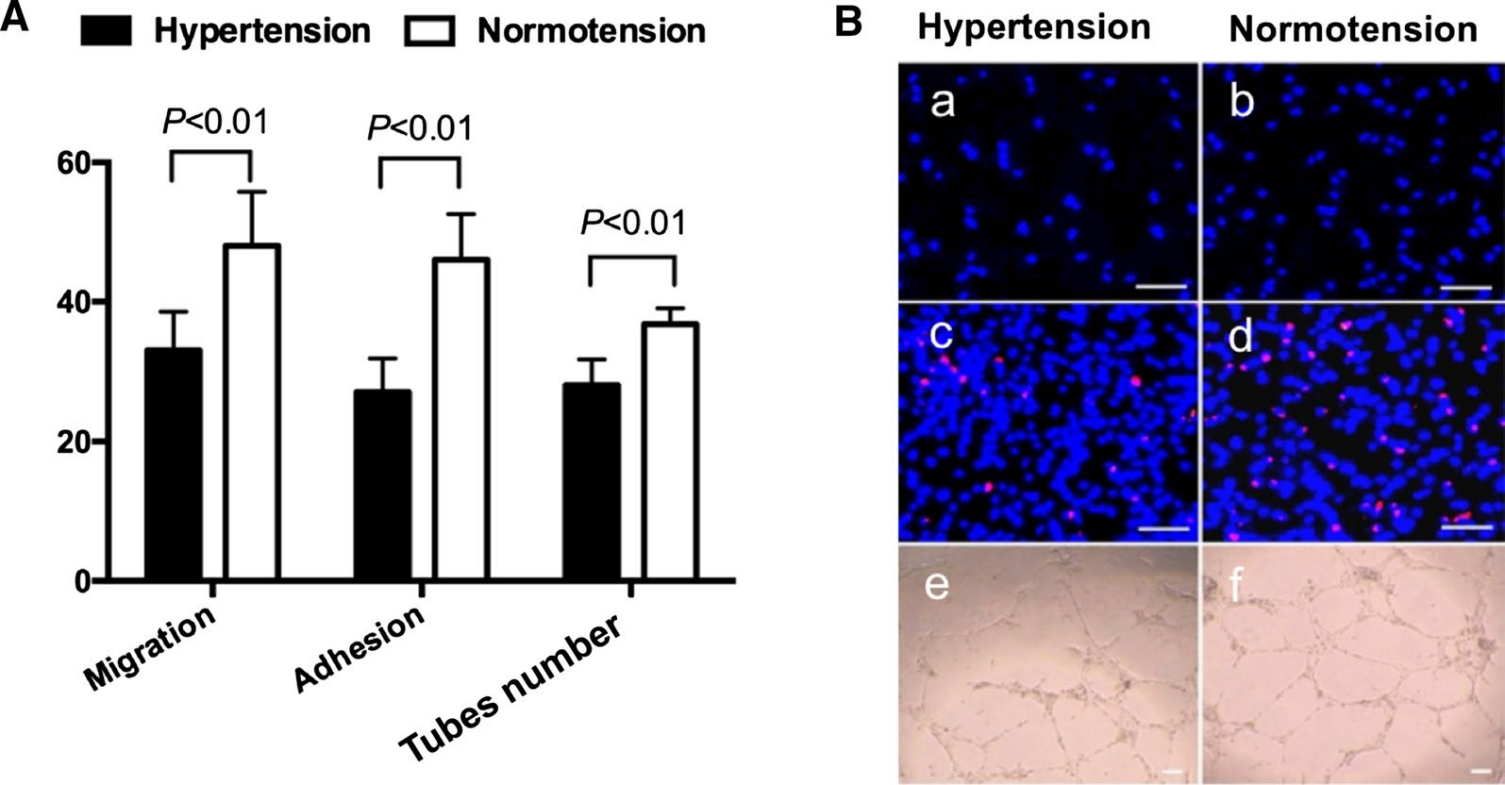
Effects of hypertension on LEPCs functions. **A** Quantification of migration, adhesion and tube formation ability of LEPCs. LEPCs functions decreased in hypertension (*P *< 0.01 vs. normotension); **B** representative photographs of LEPCs functions. a, b Representative photographs of LEPCs migration; c, d representative photographs of LEPCs adhesion; e, f representative photographs of LEPCs tube formation (magnification 100, scale bar 100 μm). Number of per hp for migration and adhesion, number of per mm^2^ for tube. *LEPCs* late endothelial cells

Angiogenesis plays a pivotal role in MVR, we investigated LEPCs functional activities from volunteers. Our results elucidated that the migration (normotension: (48 ± 7.8)/hp vs. hypertension: (33 ± 5.6)/hp, *P *< 0.001), adhesion (normotension: (46 ± 6.6)/hp vs. hypertension: (27 ± 4.9)/hp, *P *< 0.001) and tube formation abilities (normotension: (37 ± 2.5)/mm^2^ vs. hypertension: (31 ± 4.6)/mm^2^, *P *< 0.001) were significantly declined in the hypertensive group (Fig. 3).

### RCD and R-NPA were well correlated with LEPC-mediated angiogenesis in hypertension

**Fig. 4:**
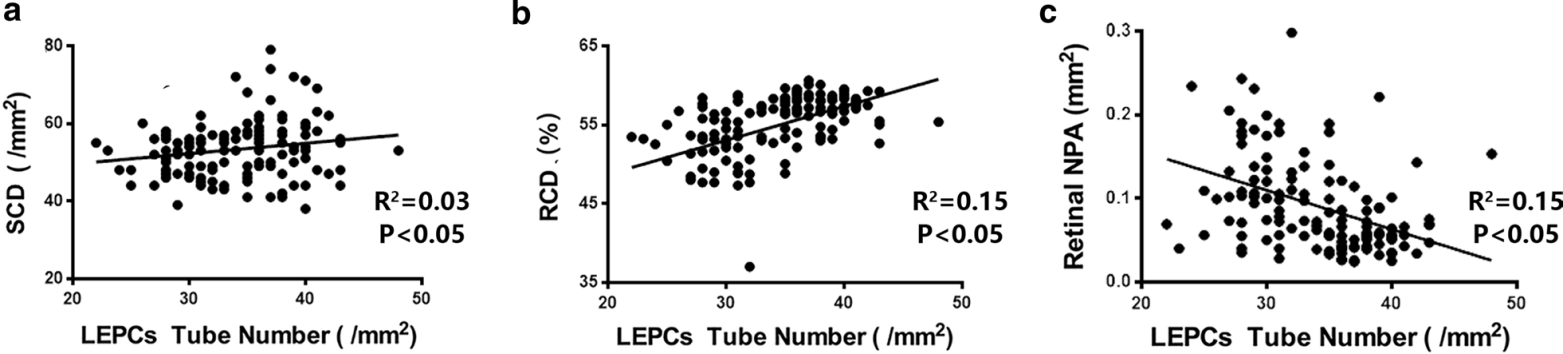
Correlation analysis of SCD, RCD/R-NPA and LEPCs tube formation. **a** Scatter plots of correlation between SCD and LEPCs tube formation; **b** scatter plots of correlation between RCD and LEPCs tube formation; **c** scatter plots of correlation between R-NPA and LEPCs tube formation. *SCD* skin capillary density, *RCD* retina capillary density, *R-NPA* retina non-perfused areas, *LEPCs* late endothelial cells

Our results of univariate regressions showed significant relation between LEPCs tube number and SCD (R = 0.186, *P *< 0.05), LEPCs tube number and RCD (R = 0.387, *P *< 0.01), LEPCs tube number and R-NPA (R = − 0.392, *P *< 0.01) (Figures and R^2^ showed in Fig. 4). After multivariate adjustments, positive correlation existed between RCD or R-NPA with LEPCs tube formation (RCD: β = 0.64, 95% CI 0.34–0.91, *P *< 0.01; R-NPA: β = − 24.67, 95% CI − 43.14 to − 4.63, *P *< 0.05) but not with SCD (β = 0.082, 95% CI 0.01–0.18, *P *= 0.085) (Table 3).

**Table 3:** Multivariate linear regression analysis for the determinants of LEPCs tube formation ability

	Unadjusted			Adjustment		
	β	95% CI	*P*	β	95% CI	P*
SCD	0.035	− 0.085 to − 0.135	0.660	0.082	0.01 to 0.18	0.085
RCD	0.252	0.108 to 0.395	< 0.001	0.64	0.34 to 0.91	< 0.001
R-NPA	− 24.277	− 37.733 to − 10.822	< 0.001	− 24.67	− 43.14 to − 4.63	0.015

P*, after adjustments for age, body mass index, dietary salt intake, mean systolic blood pressure, mean diastolic blood pressure, triglycerides, total cholesterol, low-density lipoprotein, fasting blood glucose and C reactive protein level

*SCD* skin capillary density, *RCD* retinal capillary density, *R-NPA* retinal non-perfused area

## Discussion

The major findings of the present study are as follows: (1) both NVC and OCTA can detect MVR in early hypertension. (2) OCTA results were independently related to LEPC-mediated angiogenesis and correlated better with hypertensive blood pressure level than intravital capillaroscopy. (3) In vitro functions of LEPCs were impaired and closely correlated to MVR in hypertension. To the best of our knowledge, our current study for the first time demonstrated that OCTA and intravital capillaroscopy can be both used as effective approaches to investigate MVR in mild hypertension, indicating that combination of two method would be a better way for systemic MVR detection and OCTA might be better than intravital capillaroscopy in mild hypertension. Moreover, systemic MVR in early hypertension was correlated with in vitro LEPCs dysfunctions.

MVR is an important pathophysiological process in hypertension which increases integral peripheral vascular resistance, and eventually contributes to the development of ASCVD [5, 22]. Thus, it is especially important to detect MVR as early as possible. Intravital capillaroscopy as the most traditional tool for MVR detection has been used in clinical practice since last century. Antonios et al. [23, 24] showed that intravital capillaroscopy visually exhibited the decreased number of dermal capillary in hypertensive individuals. In the present study, our results also validated that SCD decreased in hypertension compared to the controls, which is consistence with previous studies. However, MVR is a systematic change in vivo, so it stands to reason that it could be assessed in other organs. Current researches had reported retinal microcirculation impairment as the early pathophysiological change of hypertension target organ damage [25], thus it is both expedient and important to detect retinal microvasculature for systemic assessment in hypertension. However, the current retinal angiographic gold standards are invasive, and they have also been found to be unreliable because of its inter- and intra-observer variability [26], in addition to being expensive and time-consuming. Therefore, they are not ideal examinations to be carried out on a regular basis in a busy clinical setting [27, 28].

Since the SSADA technique was proposed for better refined images with a diameter less than 10 microns, quantitative numerical data and non-interfering real-time flow density map [29, 30], newest OCTA provided an advanced noninvasive way to look into the retinal capillary network, superior to former OCT with scanning laser Doppler flowmetry technique [31, 32]. It detects all levels of retinal vessels and capillaries, presenting perfect connectivity of branch and web looking patterns, which is ideal since it meets our understanding of retinal vasculature anatomy. Measurement of retinal capillary blood flow density map with three-dimensional solution, which can accurately and specifically identify the blood flow and evaluate anatomy and function of capillary bed [33]. OCTA provides accurate size, localization information and visualization of the retinal vasculature, and shows structural and functional blood flow information in tandem [34].

In this study, variables as RCD and R-NPA were first synchronously applied in hypertensive patients and showed a decrease in RCD and an increase in R-NPA. With this observation, we found not only retinal capillary structure but also blood flow was impaired in hypertension. In general, we demonstrated for the first time that structural and functional MVR were simultaneously present in retinal capillary in the early stage of hypertension. To determine its consistence, we assessed the relationship between OCTA and intravital capillaroscopy, and found both RCD and R-NPA had positive linear correlation with SCD. Our data clearly manifested the coherent trend of MVR presented in retina and skin, and documented that MVR is an integrated change in the early stage of hypertension. Previous studies revealed that chronic hypertension (more than 10 years) affects the retina OCTA parameters [35] and reduced retina microvasculature observed by OCTA correlated with higher BP [36]. However, previous studies did not focus on mild hypertension without antihypertension treatment, furthermore, only partial fundus macular microvasculature was analyzed which could not represent systemic microvasculature change in hypertension. With the combination of intravital capillaroscopy and OCTA, the present study focused on mild hypertension and showed that such combination might be more comprehensive to detect MVR in hypertension.

Although there are many factors involved in the development of hypertensive MVR, such as a lack in tissue VEGF, an extracellular matrix more resistant to peptidases, a defect in VEGF receptors, an imbalance between Ang1 and Ang2, and so on, accumulating data demonstrated that EPC-related deficient angiogenesis contributes to MVR in hypertension. Previous experiments confirmed EPCs dysfunction presented in SHR with MVR [8]. Our team provided further evidence for EPCs deficiency in hypertensive patients [37]. Additionally, LEPCs, as one specific subpopulation of EPCs, were particularly relevant with angiogenesis [9]. However, the association between LEPCs angiogenic ability and MVR in hypertension has not been reported yet. Our data showed in vitro tube formation ability of LEPCs in hypertensive patients were significantly reduced than that in normotensive subjects, in line with migration and adhesion function. Combined with SCD and OCTA data, a case could be made that deficiency in LEPCs angiogenic capacity might be related to MVR in hypertension. Notably, we for the first time found the correlation between LEPC-mediated angiogenic decline and MVR in hypertension. Based on these data, together with previous studies, it seems logical that LEPCs dysfunction might play a role in the development of hypertensive MVR, which should be further validated in future studies.

Microvascular change can be affected by age, BMI, BP, dietary salt intake, glycolipid metabolism, systemic inflammatory response and so on [38, 39]. Therefore, we analyzed the influence of these parameters on MVR examined by SCD and OCTA, and found that SCD, RCD and R-NPA were correlated well with LEPCs tube formation. Furthermore, after multivariate lineal regression analysis, RCD and R-NPA but not SCD were independently correlated with LEPCs tube formation.

Capillary rarefaction was observed in hypertensive patients with intravital capillaroscopy, compared to normotensive individuals regardless of blood pressure levels [26]. Studies demonstrated that good blood pressure control improved retinal microcirculation [40, 41], however, no data hitherto investigated the efficiency of capillary rarefaction index in relation to blood pressure levels. Our results for the first time demonstrated that RCD and R-NPA correlated with SBP and DBP, in other words, the level of pre-treatment blood pressure were related to the abnormalities of retinal microvasculature. Moreover, the AUC of RCD and R-NPA were larger than SCD both for SBP and DBP, indicating that OCTA showed better efficiency. Furthermore, the analysis indicated that OCTA might be employed as a better method to monitor the effect of anti-hypertensive management.

The current study had several limitations. First, this is a small sample size study. A study of a larger population should be performed to validate these results. Second, patients with various levels of blood pressure should be enrolled for further exploration regarding the relationship between MVR and blood pressure levels in the future. Third, since only male patients were enrolled in this study, these results should also be replicated in female population.

## Conclusion

In conclusion, our present study demonstrates for the first time that OCTA is a more precise technique for early detection of microvascular rarefaction in hypertension compared with intravital capillaroscopy, which was associated with the fall in LEPC-mediated angiogenesis. Both of OCTA and LEPCs function can help to identify hypertension-related capillary abnormality.

## Data Availability

The datasets used and analyzed during the current study are available from the corresponding author on reasonable request.

## Abbreviations

ASCVD: atherosclerotic cardiovascular diseases
MVR: microvascular rarefaction
EPCs: endothelial progenitor cells
OCTA: optical coherence tomography angiography
R-NPA: retinal non-perfusion area
RCD: retinal capillary density
EEPCs: early endothelial progenitor cells
LEPCs: late outgrowth endothelial progenitor cells
HBP: home blood pressure
VC: venous congestion
SCD: skin capillary density
OD: oculus dexter
OS: oculus sinister
SSADA: split-spectrum amplitude-decorrelation angiography
EBM-2: endothelial cell basal medium-2
CD31: cluster of differentiation 31
KDR: kinase-insert domain receptor
TNF-α: tumor necrosis factor-α
SDF-1: stromal cell-derived factor-1
BMI: body mass index
MDBP: mean diastolic blood pressure
TG: triglycerides
TC: total cholesterol
LDL: low-density lipoprotein
FBG: fasting blood glucose
ROC: receiver operating characteristic curve
AUC: area under the curve
