# Correction to: Systemic microvascular rarefaction is correlated with dysfunction of late endothelial progenitor cells in mild hypertension: a substudy of EXCAVATION-CHN1

**DOI:** 10.1186/s12967-020-02216-z

**Published:** 2020-01-16

**Authors:** Jianwen Liang, Yan Li, Long Chen, Wenhao Xia, Guifu Wu, Xinzhu Tong, Chen Su, Jiang He, Xiufang Lin, Jun Tao

**Affiliations:** 1 Department of Hypertension and Vascular Disease, The First Affiliated Hospital, Sun Yat-Sen University, Guangzhou, 510080 China; 2 Department of Cardiology, The Eighth Affiliated Hospital, Sun Yat-Sen University, Shenzhen, China; 3 Department of Cardiology, The Fifth Affiliated Hospital, Sun Yat-Sen University, Zhuhai, 519000 China; 4 Key Laboratory on Assisted Circulation, Ministry of Health, Guangzhou, 510080 China

## Correction to: J Transl Med (2019) 17:368 10.1186/s12967-019-2108-8

Upon publication of the original article [1], it was noticed that Jun Tao’s affiliation information is not complete. The full affiliation information for Jun Tao can be found below and in the complete affiliation list of this Correction article.

- Department of Hypertension and Vascular Disease, The First Affiliated Hospital, Sun Yat-Sen University, Guangzhou 510080, China.
- Key Laboratory on Assisted Circulation, Ministry of Health, Guangzhou 510080, China.
